# The 2023 Impact of Inflammatory Bowel Disease in Canada: Direct Health System and Medication Costs

**DOI:** 10.1093/jcag/gwad008

**Published:** 2023-09-05

**Authors:** M Ellen Kuenzig, Stephanie Coward, Laura E Targownik, Sanjay K Murthy, Eric I Benchimol, Joseph W Windsor, Charles N Bernstein, Alain Bitton, Jennifer L Jones, Kate Lee, Juan-Nicolás Peña-Sánchez, Noelle Rohatinsky, Sara Ghandeharian, James H B Im, Rohit Jogendran, Saketh Meka, Jake Weinstein, Tyrel Jones May, Manisha Jogendran, Sahar Tabatabavakili, Elias Hazan, Malini Hu, Jessica Amankwah Osei, Rabia Khan, Grace Wang, Mira Browne, Tal Davis, Quinn Goddard, Julia Gorospe, Kate Latos, Kate Mason, Jack Kerr, Naji Balche, Anna Sklar, Gilaad G Kaplan

**Affiliations:** SickKids Inflammatory Bowel Disease Centre, Division of Gastroenterology, Hepatology, and Nutrition, The Hospital for Sick Children, Toronto, Ontario, Canada; Child Health Evaluative Sciences, SickKids Research Institute, The Hospital for Sick Children, Toronto, Ontario, Canada; Departments of Medicine and Community Health Sciences, University of Calgary, Calgary, Alberta, Canada; Division of Gastroenterology and Hepatology, Mount Sinai Hospital, University of Toronto, Toronto, Ontario, Canada; Department of Medicine, University of Ottawa, Ottawa, Ontario, Canada; The Ottawa Hospital IBD Centre, Ottawa, Ontario, Canada; SickKids Inflammatory Bowel Disease Centre, Division of Gastroenterology, Hepatology, and Nutrition, The Hospital for Sick Children, Toronto, Ontario, Canada; Child Health Evaluative Sciences, SickKids Research Institute, The Hospital for Sick Children, Toronto, Ontario, Canada; ICES, Toronto, Ontario, Canada; Department of Paediatrics, Temerty Faculty of Medicine, University of Toronto, Toronto, Ontario, Canada; Institute of Health Policy, Management, and Evaluation, Dalla Lana School of Public Health, University of Toronto, Toronto, Ontario, Canada; Departments of Medicine and Community Health Sciences, University of Calgary, Calgary, Alberta, Canada; Department of Internal Medicine, Max Rady College of Medicine, Rady Faculty of Health Sciences, University of Manitoba, Winnipeg, Manitoba, Canada; University of Manitoba IBD Clinical and Research Centre, Winnipeg, Manitoba, Canada; Division of Gastroenterology and Hepatology, McGill University Health Centre IBD Centre, McGill University, Montréal, Quebec, Canada; Departments of Medicine, Clinical Health, and Epidemiology, Dalhousie University, Halifax, Nova Scotia, Canada; Crohn’s and Colitis Canada, Toronto, Ontario, Canada; Department of Community Health and Epidemiology, University of Saskatchewan, Saskatoon, Saskatchewan, Canada; College of Nursing, University of Saskatchewan, Saskatoon, Saskatchewan, Canada; Crohn’s and Colitis Canada, Toronto, Ontario, Canada; SickKids Inflammatory Bowel Disease Centre, Division of Gastroenterology, Hepatology, and Nutrition, The Hospital for Sick Children, Toronto, Ontario, Canada; Child Health Evaluative Sciences, SickKids Research Institute, The Hospital for Sick Children, Toronto, Ontario, Canada; Department of Medicine, University of Toronto, Toronto, Ontario, Canada; Department of Neuroscience, McGill University, Montreal, Quebec, Canada; SickKids Inflammatory Bowel Disease Centre, Division of Gastroenterology, Hepatology, and Nutrition, The Hospital for Sick Children, Toronto, Ontario, Canada; Child Health Evaluative Sciences, SickKids Research Institute, The Hospital for Sick Children, Toronto, Ontario, Canada; Division of Gastroenterology and Hepatology, University Health Network, University of Toronto, Toronto, Ontario, Canada; Department of Medicine, Queen’s University, Kingston, Ontario, Canada; Department of Gastroenterology, University of Toronto, Toronto, Ontario, Canada; Department of Internal Medicine, University of Toronto, Toronto, Ontario, Canada; Department of Medicine, University of Toronto, Toronto, Ontario, Canada; Department of Community Health and Epidemiology, University of Saskatchewan, Saskatoon, Saskatchewan, Canada; SickKids Inflammatory Bowel Disease Centre, Division of Gastroenterology, Hepatology, and Nutrition, The Hospital for Sick Children, Toronto, Ontario, Canada; Child Health Evaluative Sciences, SickKids Research Institute, The Hospital for Sick Children, Toronto, Ontario, Canada; ICES, Toronto, Ontario, Canada; Department of Medicine, University of Toronto, Toronto, Ontario, Canada; SickKids Inflammatory Bowel Disease Centre, Division of Gastroenterology, Hepatology, and Nutrition, The Hospital for Sick Children, Toronto, Ontario, Canada; Child Health Evaluative Sciences, SickKids Research Institute, The Hospital for Sick Children, Toronto, Ontario, Canada; SickKids Inflammatory Bowel Disease Centre, Division of Gastroenterology, Hepatology, and Nutrition, The Hospital for Sick Children, Toronto, Ontario, Canada; Child Health Evaluative Sciences, SickKids Research Institute, The Hospital for Sick Children, Toronto, Ontario, Canada; Departments of Medicine and Community Health Sciences, University of Calgary, Calgary, Alberta, Canada; Departments of Medicine and Community Health Sciences, University of Calgary, Calgary, Alberta, Canada; Crohn’s and Colitis Canada, Toronto, Ontario, Canada; Crohn’s and Colitis Canada, Toronto, Ontario, Canada; Department of Medicine, Memorial University of Newfoundland, St John’s Newfoundland, Canada; Departments of Medicine, Clinical Health, and Epidemiology, Dalhousie University, Halifax, Nova Scotia, Canada; Departments of Medicine, Clinical Health, and Epidemiology, Dalhousie University, Halifax, Nova Scotia, Canada; Departments of Medicine and Community Health Sciences, University of Calgary, Calgary, Alberta, Canada

**Keywords:** Crohn’s disease, Direct costs, Health services utilization, Ulcerative colitis

## Abstract

Healthcare utilization among people living with inflammatory bowel disease (IBD) in Canada has shifted from inpatient management to outpatient management; fewer people with IBD are admitted to hospitals or undergo surgery, but outpatient visits have become more frequent. Although the frequency of emergency department (ED) visits among adults and seniors with IBD decreased, the frequency of ED visits among children with IBD increased. Additionally, there is variation in the utilization of IBD health services within and between provinces and across ethnocultural and sociodemographic groups. For example, First Nations individuals with IBD are more likely to be hospitalized than the general IBD population. South Asian children with Crohn’s disease are hospitalized more often than their Caucasian peers at diagnosis, but not during follow-up. Immigrants to Canada who develop IBD have higher health services utilization, but a lower risk of surgery compared to individuals born in Canada. The total direct healthcare costs of IBD, including the cost of hospitalizations, ED visits, outpatient visits, endoscopy, cross-sectional imaging, and medications are rising rapidly. The direct health system and medication costs of IBD in Canada are estimated to be $3.33 billion in 2023, potentially ranging from $2.19 billion to $4.47 billion. This is an increase from an estimated $1.28 billion in 2018, likely due to sharp increases in the use of biologic therapy over the past two decades. In 2017, 50% of total direct healthcare costs can be attributed to biologic therapies; the proportion of total direct healthcare costs attributed to biologic therapies today is likely even greater.

**Key Points**

1. People living with IBD in Canada are admitted to hospitals and undergo surgery less often than in past decades, but these individuals have an increasing number of outpatient visits.
2. The frequency of emergency department (ED) visits among adults and seniors with IBD have decreased. Children with IBD are visiting EDs more often. Despite presenting to the ED for reasons related to their IBD, gastroenterologist consults during their ED visit and follow-up are uncommon.
3. The utilization of IBD-specific health services (outpatient visits, ED visits, and hospitalization) and medications, as well as the likelihood of undergoing surgery, vary both within and between provinces.
4. First Nations individuals with IBD are more likely to be hospitalized than the general IBD population. Additional within and between-province studies are needed to better understand healthcare and medication utilization and the risk of surgery in First Nations individuals with IBD.
5. South Asian children with Crohn’s disease are hospitalized more often at diagnosis than their Caucasian peers. However, these differences diminish during follow-up.
6. Immigrants to Canada who develop IBD have higher health services utilization, but a lower risk of surgery compared to individuals born in Canada.
7. People with IBD of low socioeconomic status are more likely to be hospitalized, undergo surgery, and require systemic steroids.
8. People with IBD living in rural areas are more likely to be hospitalized and visit the ED than people living in urban areas, but there is no difference in their risk of surgery.
9. The total direct healthcare costs of IBD—costs of hospitalizations, ED visits, outpatient visits, endoscopy, cross-sectional imaging, and medications—in Canada have risen rapidly and are estimated to be $3.33 billion in 2023 relative to market list price; these costs may be as low as $2.19 billion or as high as $4.47 billion.
10. Biologic medications contributed approximately 50% of direct healthcare costs in 2017; the proportion of total direct healthcare costs attributed to biologic therapies today is likely even greater.

## Summary of Crohn’s and Colitis Canada’s 2018 Impact of IBD: Direct Costs & Health Services Utilization

In 2018, annual healthcare costs (including healthcare utilization and medication) of IBD in Canada were estimated to be at least $1.28 billion, but this was acknowledged as likely an underestimate due to its reliance on a Manitoba costing study using data from 2006, reflecting an era where far fewer individuals were treated with biologic therapy. Direct healthcare costs for IBD are more than double that of individuals without IBD. Prescription drug costs accounted for 42% of these costs and were continuing to rise with the increasing use of biologic therapy. The introduction of biosimilars into the marketplace was anticipated to reduce these costs. People with IBD had increasing access to gastroenterologists, yet many lacked timely access, with most individuals waiting longer than six months between symptom onset and diagnosis. Seniors, individuals living in rural areas, and nonimmigrants had less access to specialist care, which was associated with better IBD outcomes. One in five adults with Crohn’s disease and one in eight adults with ulcerative colitis were hospitalized every year. Surgery rates were declining. However, within 10 years of diagnosis, one in three people with Crohn’s disease still required surgery, and one in six people with ulcerative colitis required a colectomy.

## INTRODUCTION

Canadians living with inflammatory bowel disease (IBD) have frequent interactions with the healthcare system, including being admitted to hospitals, care, undergoing diagnostic testing (endoscopy, laboratories, and imaging), and receiving ambulatory and long-term care. Nearly all Canadians are eligible for universal healthcare plans that cover the majority of the costs of these interactions. Most provinces also cover medication costs for seniors, individuals on social assistance, or those who are otherwise unable to afford needed medications. When these and other costs (e.g., costs associated with care provided by allied healthcare providers such as psychologists or dietitians) are not covered by health plans, individuals must pay out-of-pocket or purchase private insurance. These costs, borne both by individuals and the healthcare system, comprise the direct costs of IBD.

Provincial healthcare budgets are limited. Nevertheless, Canadian healthcare systems must be prepared to address the needs of a growing IBD population. Understanding the healthcare needs of people with IBD ensures Crohn’s and Colitis Canada can advocate for the resources people with IBD need. Therefore, it is critical to understand how much care is being used by Canadians with IBD, how this is changing over time, the inequities in healthcare utilization, and associated costs to provide equitable and high-quality care for people with IBD. This article outlines the direct costs of IBD borne by the Canadian health system, including medication costs (covered either through government or private pharmacare programs). For the direct costs borne by individuals (including medication costs paid out-of-pocket by a person with IBD), see Kuenzig et al. (this volume).

## HOSPITALIZATIONS

The frequency of hospitalization for IBD has steadily decreased in most Canadian regions over the past few decades, likely resulting from a combination of evolving treatment paradigms in IBD and health system pressures (Table 1) (1–6). In Ontario, hospitalizations among children with IBD declined at a slower rate than among children without IBD (IBD, average annual percentage change [AAPC]: −2.6%; 95% confidence interval [CI]: −3.3%, −1.8%; matched controls, AAPC: −4.3%; 95% CI: −5.4%, −3.3%) (3). Hospitalizations in adults with IBD also decreased in Ontario, but the introduction of biologic therapy did not alter the rate at which hospitalizations were decreasing in people with Crohn’s disease (4).

**Table 1.** Trends in hospitalization rates in Canadian people with inflammatory bowel disease

Study	Province	Years	Age group	Type of cohort	Definition of hospitalization	Type of IBD	Hospitalization rates	Time trend (95% CI)
Coward et al. (5)	Alberta	2002–2014	All ages	Prevalent	IBD-specific (admission directly resulting from IBD)	IBD	2002: 16.8 per 100 people (95% CI: 16.4, 17.2) 2014: 8.7 per 100 people (95% CI: 8.5, 9.0)	AAPC: −3.77% (−4.63, −3.08)
					IBD-related (admission for IBD or a symptom or comorbidity associated with IBD)	IBD	2002: 22.6 per 100 people (95% CI: 22.1, 23.1) 2014: 13.4 per 100 people (95% CI: 13.2, 13.7)	AAPC: −3.09% (−3.65, −2.62)
					All reasons	IBD	2002: 35.3 per 100 people (95% CI: 34.7, 35.9) 2014: 24.9 per 100 people (95% CI: 24.5, 25.2)	AAPC: −2.12% (−2.31, −1.93)
Targownik et al. (1)	Manitoba	2005–2015	All ages	Prevalent	IBD-attributable (difference in the total number of hospitalizations when comparing people with and without IBD)	CD	2005: 19.08 per 100 PY 2015: 11.75 per 100 PY	AAPC: −3.0% (−3.7, −2.3)
						UC	2005: 8.11 per 100 PY 2015: 7.89 per 100 PY	AAPC: 0.3% (−0.5, 1.1)
					Urgent IBD-specific (hospitalizations including an overnight stay with a most-responsible diagnosis of CD or UC)	CD	2005: 6.21 per 100 PY 2015: 3.19 per 100 PY	AAPC: −6.0% (−7.5, −4.3)
						UC	2005: 2.63 per 100 PY 2015: 1.96 per 100 PY	AAPC: 0.4% (−2.0, 2.7)
Dheri et al. (3)	Ontario	1994–2012	Paediatrics (<18 years)	Incident	All reasons	IBD	—	AAPC: −2.6% (−3.3, −1.8)
						CD	—	—
						UC	—	—
					IBD-specific (hospitalizations with a most responsible diagnosis of IBD)	IBD	—	AAPC: −2.5% (−3.2, −1.8)
						CD	—	AAPC: −3.0% (−3.8, −2.1)
						UC	—	AAPC: −1.1% (−2.5, 0.003)
					IBD-related (hospitalizations with IBD or its signs, symptoms, and extra-intestinal manifestations as a most responsible diagnosis)	IBD	—	AAPC: −1.7% (−2.4, −1.0)
						CD	—	AAPC: −3.0% (−3.8, −2.1)
						UC	—	AAPC: −1.4% (−2.8, −0.0004)
Murthy et al. (4)	Ontario	1995–2012	Adults (>18 years)	Prevalent	IBD-related (visits with either CD or UC as the most responsible comorbid, or primary interservice or interhospital transfer diagnosis)	CD	—	Pre-infliximab: OR: ^†^0.980 (0.975, 0.985) Post-infliximab: OR: ^†^1.00 (0.998, 1.01)
						UC	—	Pre-infliximab: OR: ^†^0.976 (0.973, 0.979) Post-infliximab: OR: ^†^1.22 (1.07, 1.39)
Rahman et al. (2)	Ontario	2003–2014	All ages	Prevalent	CD was the most responsible diagnosis for the admission	CD	2003: 154 (95% CI: 150, 159) per 1000 2014: 104 (95% CI: 101, 107) per 1000	32.4% decrease over the course of the study
Verdon et al. (6)	Québec	1996–2015	Not stated	Incident	IBD-related	CD	1996–2010: 19%^*^ 2011–2015: 45%^*^	—
						UC	1996–2010: 21%^*^ 2011–2015: 44%^*^	—

Abbreviations: AAPC, Average annual percentage change; CD, Crohn’s disease; CI, Confidence interval; IBD, Inflammatory bowel disease; OR, Odds ratio; PY, Person-years; UC, Ulcerative colitis.

^*^Hospitalization rates among biologic users only.

^†^Quarter analyzed as a continuous variable; odds ratio (OR) compares the odds of hospitalization per quarter change in time.

### Costs of Hospitalization

Hospitalization costs directly attributable to Crohn’s disease decreased between 2005 and 2015, from $2,565 to $1,426 per person with Crohn’s disease per year; hospitalization costs attributable to ulcerative colitis remained steady (costs in 2015 Canadian dollars [CAD]) (1). In Alberta, British Columbia, and Saskatchewan, hospitalization costs ranged from $2,372 to $4,472 per person with IBD per year (Alberta and British Columbia cost in 2020 CAD; Saskatchewan costs in 2013 CAD) (7,8). Hospitalization costs accounted for 35% to 45% of health system costs in 2009, decreasing to 22% to 28% in 2015 (7). In these studies, hospitalization costs were averaged over all people with IBD, regardless of whether they were hospitalized. Costs of inpatient admissions for Manitoba children with IBD decreased from $365,252 in 2004 to $61,600 in 2017 (*P* < 0.01); per-person hospitalization costs were not reported (costs in 2018 CAD) (9). The authors attributed this decrease in hospitalization costs to a reduction in IBD-related surgeries.

## SURGERIES

When medications are not working or IBD-related complications occur (e.g., strictures, fistulae, abscesses, or colorectal cancer), individuals with IBD may require surgery. The type and location of surgery depends on disease location and phenotype. The most common surgery in Crohn’s disease is an intestinal resection (2), most often an ileocecal resection (10). People with ulcerative colitis may require a colectomy with or without the creation of an ileal pouch-anal anastomosis (typically a J-pouch in more recent years). Within five years of diagnosis, 12% (95% CI: 8%, 15%) of children with Crohn’s disease required an intestinal resection and 12% (95% CI: 10%, 14%) of children with ulcerative colitis required a colectomy (11,12). One-third of Ontario seniors required intestinal resection within five years of a Crohn’s disease diagnosis; one in five Ontario seniors required a colectomy within five years of an ulcerative colitis diagnosis (11).

As IBD management has evolved, surgery rates have decreased (Table 2) (1–4,9,10,13). However, these declining trends existed before the introduction of biologics, and their introduction into the market has not appreciably changed the rate at which surgeries are decreasing (4).

**Table 2.** Trends in surgery rates in Canadian people with Crohn’s disease and ulcerative colitis

Study	Province	Years	Age group	Type of cohort	Type of IBD	Surgery rates	Time trend (95% CI)
Dittrich et al. (10)	Alberta (Edmonton)	1996–2013	Adults (≥18 years)	Prevalent	CD	1996: 5.8 per 100 patients 2013: 1.4 per 100 patients	AAPC: −8.4% (−9.6, −7.3)
El-Matary et al. (9)	Manitoba	1995–2017	Paediatrics	Incident	IBD	1995–2003: 5.2 per 100 PY 2004–2017: 1.8 per 100 PY	RR: 0.34 (0.20, 0.59)
Targownik et al. (1)	Manitoba	2005–2015	All ages	Prevalent	CD	2005: 1.88 per 100 PY 2015: 1.27 per 100 PY	AAPC: −3.6% (−6.0, −1.2)
				Prevalent	UC	2005: 0.85 per 100 PY 2015: 0.83 per 100 PY	AAPC: 1.7% (−2.0, 5.4)
Dheri et al. (3)	Ontario	1994–2012	Paediatrics (<18 years)	Incident	CD	**—**	AAPC: −6.0% (−7.3, −4.6)
					UC	**—**	AAPC: −3.0% (−5.2, −0.7)
Murthy et al. (4)	Ontario	1995–2012	Adults (>18 years)	Prevalent	CD	—	Pre-infliximab: OR:^†^ 0.984 (0.975, 0.99) Post-infliximab: OR:^†^ 1.10 (0.81, 1.50)
					UC	**—**	Pre-infliximab: OR:^†^ 0.993 (0.975, 1.01) Post-infliximab: OR:^†^ 0.933 (0.540, 1.61)
Rahman et al. (2)	Ontario	2003–2014	All ages	Prevalent	CD	All inpatient surgeries: 2003: 53 (95% CI: 50, 55) per 1000 2014: 32 (95% CI: 30, 34) per 1000 Intestinal resections: 2003: 41 (95% CI: 39, 43) per 1000 2014: 23 (95% CI: 22, 25) per 1000 All outpatient surgeries:* 2003: 8 (95% CI: 7, 9) per 1000 2014: 12 (95% CI: 10, 13) per 1000	All inpatient surgeries: 39.6% decrease over the course of the study Intestinal resections: 44% decrease over the course of the study
Khalil et al. (13)	Québec	1998–2011	Not stated	Prevalent	UC	1998–2004: 36 per 1000 PY 2005–2011: 30 per 1,000 PY	HR:^‡^ 0.81 (0.70, 0.95)
Verdon et al. (6)	Québec	1996–2015	Not stated	Incident (proportion with surgery within five years of diagnosis)	CD	1^st^ surgeries: 1996–2010: 8% 2011–2015: 15% 2^nd^ surgeries: 1996–2010: 18% 2011–2015: 21%	**—**
					UC	1996–2010: 6% 2011–2015: 10%	**—**

Abbreviations: AAPC, Average annual percentage change; , Confidence interval; IBD, Inflammatory bowel disease; OR, Odds ratio; PY, Person-years; RR, Relative risk; UC, Ulcerative colitis.

^*^Includes stricture dilations and stricturoplasty (61.6% in 2014) and fistula or perianal surgeries (36.9% in 2014).

^†^Quarter analyzed as a continuous variable; odds ratio (OR) compares the odds of hospitalization per quarter change in time.

^‡^Comparing across eras.

## EMERGENCY DEPARTMENT VISITS

The frequency of Crohn’s disease-related emergency department (ED) visits decreased from 141 (95% CI: 137, 146) in 2003 to 101 (95% CI: 99, 104) in 2014 per 1,000 prevalent individuals with Crohn’s disease (2). Trends were similar in adults and seniors (2). ED visits among children increased for IBD-related reasons (AAPC: 1.5%; 95% CI: 0.7%, 2.3%) and all causes (AAPC: 1.0%; 95% CI: 0.1%, 1.9%) (3), while the frequency of all-cause ED visits among children without IBD remained stable (AAPC: −0.2%; 95% CI: −0.6%, 0.2%) (3). The average cost of an ED visit in Winnipeg, Manitoba was $650 (2017 CAD); costs were driven by imaging and specialist consults (14).

Sixty percent of ED visits among people with IBD occur outside of traditional office hours (14). One-third of people visiting the ED are admitted to the hospital (15). Those not admitted to the hospital averaged 5.2 IBD-related ED visits over a three-year period; half had seen their gastroenterologist in the past year (15). Gastroenterologists were only consulted during 19% of first IBD-related ED visits (15), but the likelihood of seeing a gastroenterologist increased with repeat visits (14). People seen by a gastroenterologist during their ED visit were significantly more likely to have a follow-ups with a gastroenterologist following discharge from the ED (79% *vs.* 27%) (15).

## OUTPATIENT CARE

On average, people with IBD have an additional 3.7 outpatient visits per person per year compared to people without IBD (16). Between 2005 and 2015, the number of IBD-attributable outpatient visits in Manitoba increased from 573 to 681 per 100 person-years among individuals with Crohn’s disease and from 376 to 522 per 100 person-years among individuals with ulcerative colitis (1). The frequency of IBD-specific outpatient visits was stable among Ontario children diagnosed with IBD before 2005 (AAPC: 0.6%; 95% CI: −0.04%, 1.2%), but increased by 4.0% (95% CI: 3.1%, 4.9%) per year among those diagnosed since 2005 (3). Since 2005, the frequency of all-cause outpatient visits for children without IBD decreased by 0.7% per year (95% CI: 0.0002%, 1.4%) but increased by 2.1% per year (95% CI:1.2%, 3.0%) among children with IBD. Each year, an individual with IBD accrued an average of $1,663 (British Columbia) and $1,898 (Alberta) in costs related to physician visits; these costs remained stable over time (costs in 2020 CAD) (7).

### Impact of Specialist Care on IBD Outcomes

While people with IBD living in rural areas were more likely to visit the ED than those living in urban areas, this association was not mediated by geographic differences in access to specialist care (17). People with IBD living in an Ontario Local Health Integration Network—with fewer gastroenterologists per capita and fewer people receiving regular follow-up care with a gastroenterologist—were more likely to visit the ED (18). Among Ontario seniors, having a gastroenterologist as the primary provider of IBD care was not associated with IBD-specific ED visits, hospitalizations, or intestinal resection in Crohn’s disease, even when controlling for community-level availability of gastroenterologists (11). Ontario seniors whose ulcerative colitis care was managed by a gastroenterologist were less likely to require a colectomy (odds ratio [OR]: 0.78; 95% CI: 0.63, 0.97) and more likely to take immunomodulators (OR: 1.69; 95% CI: 1.41, 2.02) (11).

## ENDOSCOPY AND NON-INVASIVE IMAGING

People with IBD are significantly more likely to undergo abdominal imaging (19). One-third of people with IBD had ≥1 abdominal plain film, barium enema, or abdominal/pelvic CT; <10% of matched controls had ever undergone these procedures. Almost 40% of people with IBD had an abdominal ultrasound compared to 18% of matched controls while 11.5% of people with IBD and 1.1% of controls had an abdominal/pelvic MRI. These estimates are derived from health administrative data, which currently do not provide the information needed to describe the recent increases in point-of-care bowel ultrasounds within outpatient IBD clinics. Additional information on the utilization of point-of-care bowel ultrasounds and barriers to universal access to this imaging modality is provided in Mathias et al. (this volume).

The proportion of people with an abdominal CT for Crohn’s disease increased by 11% (95% CI: 9%, 11%) per year between 1999 and 2007, then decreased by 2% (95% CI: 2%, 3%) per year until 2017 (19). The proportion of people with an abdominal MRI for Crohn’s disease increased by 25% (95% CI: 21%, 29%) per year between 1999 and 2007 and by 34% (95% CI: 29%, 40%) per year between 2007 and 2012. The use of abdominal MRI continued to increase after 2012, but at a slower pace (6% per year, 95% CI: 3%, 10%). The use of abdominal ultrasounds decreased by 2% per year (95% CI: 1%, 3%) between 1999 and 2007, then increased by 3% per year (95% CI: 2%, 4%). Trends in abdominal CT and ultrasound for ulcerative colitis mirrored those of Crohn’s disease. Abdominal MRI was much less common in ulcerative colitis and its use increased at a slower pace than in Crohn’s disease.

## MEDICATION USE

Using provincial health administrative data to describe how many people in a province are taking a type of therapy can be challenging due to differences in how these data are captured. In Alberta and Manitoba, information is available on all medications filled at an outpatient pharmacy in the province. In others (e.g., Ontario and Québec), data are limited to ­outpatient prescriptions for those eligible for provincial drug plans; in these provinces, individuals ≥65 years of age and those on social assistance are eligible for provincial drug plans. Individuals who need assistance to cover costs associated with expensive medications (e.g., biologics) may also be eligible for provincial drug plans, but off-label utilization of biologics is often funded by compassionate use programs of pharmaceutical companies, and therefore the dose/interval of their use may not be accurately reflected in provincial drug databases.

### Corticosteroids

Among individuals eligible for provincial drug coverage in Québec, there has been no change in corticosteroid utilization (6), while a small decrease in corticosteroid use has been noted in people with Crohn’s disease in Edmonton, Alberta (AAPC: −1.9%; 95% CI: −3.0%, −0.7%) (10).

### Biologics

Multiple Canadian provinces have reported the rapidly increasing use of biologics to treat IBD. Between 1996 and 2013, biologic use increased by 36.2% (95% CI: 31.3%, 41.5%) per year among individuals with Crohn’s disease in Edmonton, Alberta (10). Among those eligible for drug coverage in Ontario, infliximab utilization increased from 2.2% (95% CI: 1.0%, 3.4%) to 16.2% (95% CI: 14.1%, 18.4%) between 2003 and 2017; adalimumab utilization increased from 0% to 2.5% (95% CI: 1.7%, 3.4%) during the same time frame (2). In Québec, 4% of individuals diagnosed with Crohn’s disease before 2011 and eligible for provincial drug coverage received a biologic within five years of diagnosis compared to 16% of those diagnosed after 2010 (6). Biologic usage among people with ulcerative colitis in Québec similarly increased from 2% among those diagnosed before 2011 compared to 13% among those diagnosed after 2010 (6). This rapid increase in biologic use likely results from an increasing recognition of the importance of shifting treatment paradigms, which include the earlier introduction of biologic therapy and aiming for short- and long-term control of both clinical and objective markers of disease activity (e.g., endoscopic or cross-sectional imaging and fecal calprotectin). The evolving paradigm of IBD management is outlined in Murthy et al. (this volume).

### Immunomodulators

Among people in Québec with IBD eligible for provincial drug coverage, thiopurine use significantly increased when comparing the pre-biologic (before 2011) and biologic eras (after 2010) (Crohn’s disease, 21% to 24%; *P* < 0.001; ulcerative colitis, 13% to 16%, *P* < 0.001); the proportion of people on methotrexate also increased but was much smaller (6). Between 1996 and 2013, immunomodulator uses increased by 5.0% per year (95% CI: 2.7%, 7.4%) among people with Crohn’s disease in Edmonton, Alberta (10). The increasing use of immunomodulators is likely due to their use in combination therapy with a biologic, rather than the use of immunomodulators on their own.

### 5-Aminosalicylates (5-ASA)

Fewer people with Crohn’s disease in Québec diagnosed after 2010 filled prescriptions for 5-ASAs as compared to the individuals diagnosed in 2010 or earlier (21% vs. 33%) (6). Forty percent of people with ulcerative colitis used 5-ASAs, and rates did not change over the course of the study.

### Medication Costs

Medications accounted for approximately 50% of all IBD direct healthcare costs in 2016 (7) and are expected to make up an even larger proportion of total costs as the number of individuals on biologics increases and newer biologics become available. Total direct healthcare costs (including medication costs) in the year following a biologic start in Manitoba were $42,876 per person per year, compared to $5,153 in the year prior to the biologic start; costs were sustained among those staying on their biologic (costs are in 2015 CAD) (20). Medication costs in Saskatchewan significantly increased from an average of $660 (95% CI: $595, $732) per person per year in 1999/00 to $6,530 (95% CI: $6,024, $7,078) in 2016/17 (costs are in 2013 CAD) (8). Similar increases in cost were reported in Manitoba between 2005 and 2015, where costs of antiTNFs increased from $181 per person per year to $5,720 per person per year (costs are in 2015 CAD) (1). These costs were averaged among the total population of people with IBD and were not specific to those receiving antiTNF therapy.

AntiTNF therapies dispensed for any reason (e.g., IBD, rheumatoid arthritis, psoriasis), cost Canadian taxpayers nearly $1 billion in 2019; this cost estimate accounted only for antiTNFs taken by those eligible for provincial drug programs (3.9% of biologics dispensed) (21). The introduction of biosimilars for infliximab (2015) and adalimumab (2021) may help stem the increasing costs of biologic therapy. However, the less expensive biosimilars have not been widely adopted. In 2019, only 15.5% of antiTNFs dispensed for any indication were biosimilar agents (21). Some provinces have introduced policies to encourage biosimilar use, including requiring that all new antiTNFs started be biosimilars and/or instituting nonmedical mandatory switching of people from an originator biologic to a biosimilar. Nonmedical mandatory switch policies would result in cost savings due to decreased medication expenditures (21,22). However, these cost savings would be accompanied by a loss of effectiveness in 84% of simulations, corresponding to a loss of 0.13 (95% CI: 0.07, 0.16) quality-adjusted life years over a five-year time frame (23). The estimated cost savings over five years of $46,194 (95% CI: $42,420, $50,455) of a nonmedical switch program does not account for indirect costs (e.g., decreased workplace productivity) that would accompany a decrease in effectiveness. Additionally, potential cost savings from antiTNF biosimilars will be partially offset by increasing the use of newer classes of biologic therapy that are at least as expensive as antiTNF agents. For additional information about the efficacy and safety of biosimilars, see Murthy et al. (this volume).

## VARIATIONS IN HEALTH SERVICES, MEDICATION UTILIZATION AND SURGERY

While all Canadians are eligible for universal healthcare coverage, there remains significant variation in healthcare utilization among Canadians living with IBD. Two studies have specifically investigated this variation in healthcare utilization. The first compared variation in health services utilization and surgery within the first five years following IBD diagnosis among children treated at tertiary paediatric centres in Alberta, Manitoba, Nova Scotia, and Ontario (12). The second compared health services, medication utilization, and surgery among seniors in Ontario across healthcare networks (11).

The proportion of children with IBD who were hospitalized varied significantly across paediatric centres (*I*^2^: 84%, *τ*: 0.1556) (12), but the risk of hospitalization among seniors was similar across healthcare networks (median odds ratio [MOR]: 1.0) (11). The risk of colectomy among children with ulcerative colitis did not vary across paediatric centres (*I*^2^: 0%, *τ*: 0); the risk of intestinal resection in children with Crohn’s disease did vary (*I*^2^: 81%, *τ*: 0.042) (12). The opposite was true in Ontario seniors: There was significant variation in the risk of colectomy among seniors with ulcerative colitis (MOR: 1.37, *P* = 0.01) but not in the risk of intestinal resection among seniors with Crohn’s disease (MOR: 1.32, *P* = 0.08) (11).

Children with IBD varied in the frequency at which they visited an ED (*I*^2^: 99%, *τ*: 1.33), largely attributable to between-province differences (12). Among Ontario seniors with IBD, there was variation in the likelihood that they visited an ED at the time of IBD diagnosis (MOR: 1.30, *P* = 0.023), but not within five years (MOR: 1.02, *P* = 0.49) (11).

The utilization of some medications but not others varied among Ontario seniors treated in different healthcare networks. The use of both corticosteroids and immunomodulators within five years of IBD diagnosis varied (corticosteroids, MOR: 1.26, *P* = 0.006; immunomodulators, MOR: 1.46, *P* = 0.001) (11). In contrast, there was no significant variation in the use of biologics among seniors (MOR 1.15, *P* = 0.34) (11).

While we cannot determine if variability in health service, medication utilization and surgery results from differential access to the healthcare system or other factors that shift an individual’s likelihood of seeking healthcare, it is important that we recognize that there are differences in access to and provision of care that may impact long-term outcomes. Canadian studies have aimed to compare health services and medication utilization across sociodemographic (defined by socioeconomic status and rural/urban residence) and ethnocultural groups (including First Nations individuals, those of South Asian descent, and immigrants to Canada).

### Socioeconomic Differences

A Manitoba study used health administrative data to compare health services utilization among people with IBD classified as having low socioeconomic status compared to those of higher socioeconomic status (24). In this study, individuals with low socioeconomic status met at least one of the following three criteria: (1) received employment and income assistance; (2) registered with child and family services or had a child registered with child and family services; or (3) lived in an area defined as being in the highest quintile based on the Socioeconomic Factor Index version 2. Individuals with IBD classified as having low socioeconomic status were more likely to be hospitalized (any reason, relative risk [RR]: 1.38; 95% CI: 1.31, 1.44; IBD-specific reasons, RR: 1.28; 95% CI: 1.18, 1.39). People classified as having low socioeconomic status also had an increased risk of being admitted to an intensive care unit (RR: 1.94; 95% CI: 1.65, 2.27). Low socioeconomic status was associated with an increased risk of surgery among prevalent IBD patients (hazard ratio [HR]: 1.11; 95% CI: 1.00, 1.23) but not incident IBD patients (HR: 1.12; 95% CI: 0.99, 1.27). People with IBD and low socioeconomic status had more all-cause outpatient visits (RR: 1.10; 95% CI: 1.06, 1.13) but not IBD-specific outpatient visits (RR: 1.04; 95% CI: 0.98, 1.10); socioeconomic status was not associated with seeing a gastroenterologist (RR: 1.02; 95% CI: 0.94, 1.10). Low socioeconomic status was associated with increased use of corticosteroids (RR: 1.11; 95% CI: 1.01, 1.21) but not biologic use (RR: 1.03; 95% CI: 0.91, 1.16).

### Individuals Living in Rural and Urban Areas

Data from multiple provinces have described differences in healthcare utilization among people with IBD living in rural and urban regions (17,25). People with IBD living in rural regions had more IBD-specific hospitalizations than those in urban regions (Alberta/Ontario/Manitoba combined, incidence rate ratio [IRR]: 1.17; 95% CI: 1.02, 1.34; Saskatchewan, IRR: 1.22; 95% CI: 1.09, 1.37) (17,25). People living in urban and rural areas had a similar risks of first intestinal resection for Crohn’s disease and colectomy for ulcerative colitis (17,25), but the association with needing a second surgery among people with Crohn’s disease varied by province (Ontario, OR: 1.55; 95% CI: 1.16, 2.08; Alberta, OR: 1.14; 95% CI: 0.73, 1.80; Manitoba, OR: 0.80; 95% CI: 0.39, 1.66) (17). People with IBD living in rural areas present to ED significantly more often than those living in urban areas for IBD-specific reasons (IRR: 1.53; 95% CI: 1.42, 1.65) (17). Only unscheduled visits to ED were counted; any scheduled visits (e.g., for biologic infusion) did not contribute to the increased number of visits among those living in rural areas. There was no difference in the time to endoscopy for people living with IBD in rural versus urban areas in Saskatchewan (HR: 0.94; 95% CI: 0.87, 1.00); however, people living in rural areas had fewer endoscopies during the course of the study (IRR: 0.92; 95% CI: 0.87, 0.98) (25).

The frequency of IBD-specific outpatient visits was similar among people living in urban and rural areas of Alberta, Manitoba, and Ontario (IRR: 0.99; 95% CI: 0.88, 1.01) (17). Furthermore, individuals living in rural areas were less likely to have ever seen a gastroenterologist for IBD-specific reasons (Alberta, Manitoba, and Ontario, OR: 0.46; 95% CI: 0.32, 0.65; Saskatchewan, OR: 0.60; 95% CI: 0.51, 0.70), and they had fewer IBD-specific gastroenterology visits (Saskatchewan, IRR: 0.89; 95% CI: 0.83, 0.95) (17,25). The differences in the likelihood of having seen a gastroenterologist varied by age: No differences were noted in children with IBD (<10 years at diagnosis, OR: 0.97; 95% CI: 0.77, 1.20; 10–18 years at diagnosis, OR: 0.70; 95% CI: 0.47, 1.04) and the largest differences were seen in individuals ≥65 years at diagnosis (OR: 0.35; 95% CI: 0.26, 0.46).

People living with IBD in rural and urban areas of Saskatchewan were equally likely to be treated with biologic and immunomodulator therapies (biologic, HR: 0.89; 95% CI: 0.78, 1.01; immunomodulator, HR: 0.93; 95% CI: 0.84, 1.03) (25). Similarly, there were no differences in the likelihood of becoming steroid dependent among these two groups (OR: 0.94; 95% CI: 0.79, 1.12) (25). Rural living for those living with Crohn’s disease, but not those living with ulcerative colitis in Saskatchewan were more likely to be prescribed 5-ASA (Crohn’s disease, HR: 1.13; 95% CI: 1.02, 1.26; ulcerative colitis, HR: 1.06; 95% CI: 0.97, 1.16) (25).

### First Nations Individuals

A study using health administrative data in Saskatchewan compared health services and medication utilization among First Nations individuals with IBD not living on reserves to the general IBD population (26). After adjusting for rural/urban residence, First Nations individuals with IBD in Saskatchewan were more likely than the general IBD population to be hospitalized for IBD-specific reasons (HR: 1.33; 95% CI: 1.01, 1.75). First Nations individuals living with IBD might have a different risk of surgery compared to the general IBD population (Crohn’s disease, HR: 0.93; 95% CI: 0.51, 1.70; ulcerative colitis, HR: 1.30; 95% CI: 0.83, 2.05). Endoscopy rates were similar among First Nations individuals and people from the general population when adjusting for rural/urban residence (HR: 1.14; 95% CI: 0.92, 1.41) but not in the unadjusted model (HR: 1.25; 95% CI: 1.01, 1.54).

There might be differences in biologic and immunomodulator therapy between First Nations individuals with IBD and people with IBD from the general population (biologic therapy unadjusted for rural/urban residence, HR: 0.58; 95% CI: 0.34, 0.99; biologic therapy adjusted for rural/urban residence, HR: 0.65; 95% CI: 0.38, 1.11; immunomodulator therapy adjusted for rural/urban residence, HR: 0.79; 95% CI: 0.55, 1.55) (26). First Nations individuals were less likely to be prescribed 5-ASA (HR: 0.56; 95% CI: 0.45, 0.71); this was consistent for Crohn’s disease and ulcerative colitis (26). Because of the relatively small numbers of First Nations individuals living with IBD, these estimates are imprecise and further studies within and across provinces are needed. Unfortunately, there is also a paucity of research addressing IBD among Indigenous communities in Canada more broadly.

### South Asian Individuals with IBD

The risk of hospitalization/surgery was compared among South Asian and non-Jewish Caucasian children using data from the Canadian Children Inflammatory Bowel Disease Network (CIDsCaNN)—a cohort study of children newly diagnosed with IBD across 12 Canadian paediatric hospitals (27). South Asian children with Crohn’s disease were more likely to be hospitalized at diagnosis (OR: 3.30; 95% CI: 1.36, 8.03); the odds of hospitalization were similar among South Asian and Caucasian children with ulcerative colitis (OR: 1.09; 95% CI: 0.51, 2.30). In the 18 months following diagnosis, there were no differences in the risk of hospitalization across ethnic groups (Crohn’s disease, HR: 1.30; 95% CI: 0.57, 2.98; ulcerative colitis, HR: 0.80; 95% CI: 0.40, 1.60). There were no differences in initial induction or maintenance therapy when comparing South Asians and Caucasians. However, South Asian children with Crohn’s disease were more likely to receive a steroid course during follow-up (HR: 3.41; 95% CI: 1.11, 10.5); no differences were noted among children with ulcerative colitis (HR: 1.46; 95% CI: 0.78, 2.73).

### Immigrants to Canada

Health services utilization among immigrants to Canada was compared to Canadian-born individuals using Ontario health administrative data linked to data from Immigration, Refugees, and Citizenship Canada (28). Canadian immigrants had more IBD-specific outpatient visits (IRR: 1.24; 95% CI: 1.15, 1.33), ED visits (IRR: 1.57; 95% CI: 1.30, 1.91), and hospitalizations (IRR: 1.19; 95% CI: 1.02, 1.40). Immigrants to Canada were also more likely to have seen a gastroenterologist for IBD-specific care (OR: 1.37; 95% CI: 1.34, 1.40). Immigrants to Canada were less likely to require surgery (Crohn’s disease, HR: 0.66; 95% CI: 0.43, 0.99; ulcerative colitis, HR: 0.52; 95% CI: 0.31, 0.87). When adjusting for increased specialist care among immigrants to Canada, the association between immigration status and surgery was no longer significant in Crohn’s disease (HR: 0.87; 95% CI: 0.57, 1.31); immigrants to Canada with ulcerative colitis were still less likely to require surgery (HR: 0.56; 96% CI: 0.33, 0.93). These findings were generally similar regardless of region of origin.

## ESTIMATING THE TOTAL DIRECT COSTS OF IBD IN CANADA

A Manitoba study reported significantly increasing total direct healthcare costs (including the costs of outpatient care, hospitalizations, surgeries, and medication) among people with IBD, from $3,354 per person per year in 2005 to $7,801 in 2015 (costs in 2015 CAD) (1). The increase in costs has been even greater for paediatric IBD, increasing from $1811 per person per year in 2004 to $14,792 per person per year in 2017 (costs in 2020 CAD); the cost of caring for children without IBD did not change over the course of the study (9). The average direct costs among children in Ontario diagnosed at <17 years of age between 2013 and 2019 was $14,451 (SD: 14,665) in the first year following diagnosis (29). People with higher healthcare costs were more likely to have Crohn’s disease, have an ED visit at the time of diagnosis, be of older age at diagnosis, and have one or more healthcare encounters for a mental health concern. A Saskatchewan study reported an annual increase of 9.5% (95% CI: 8.9, 10.1) in total direct costs, increasing from $1,879 (95% CI: $1,686, $2,093) per person in 1999 to $7,815 (95% CI: $6,733, $7,667) per person in 2016 (costs in 2013 CAD) (8).

In a study combining health administrative data from Alberta, British Columbia, and Manitoba, the total estimated direct costs for individuals with IBD were $10,336 (95% CI: $6,803, $13,869) in 2016/17, a significant increase from $7,000 (95% CI: $5,389, $8,610) in 2009/10 (costs in 2020 CAD) (7). Extrapolating the 2016/17 per person cost from these three Western provinces to the estimated 332,598 Canadians living with IBD, the total direct costs across Canada in 2023 are estimated to be $3.33 billion. Based on the confidence interval surrounding the estimated cost per person, these costs may be as low as $2.19 billion or as high as $4.74 billion. This estimate includes health system costs, as well as medication costs. However, medication costs are difficult to estimate because the listed price of biologics is not necessarily the negotiated cost paid for by public and private drug insurance. This calculation used the listed price of biologic therapies, which may not represent the actual cost spent by payers (through private health insurance plans, government programs, or out-of-pocket by individuals themselves).

In 2018, we estimated the direct costs of IBD in Canada to be at least $1.28 billion (30), based on an estimated 270,000 people living with IBD in Canada and an estimated per person cost was based on Manitoba data from 2006 ($4,731 per person per year after adjusting for inflation to 2018 CAD) (31). This was the most conservative estimate available at the time. A Québec study estimated the annual per person cost of ulcerative colitis between 2005 and 2011 to be more than double that of the Manitoba study ($8,900; $9,690 after adjusting for inflation to 2018 CAD) (32). Had the Québec study been used to estimate total costs, the cost of IBD in 2018 would have been estimated to be $2.62 billion. Both the Manitoba and Québec studies provide data from a time when antiTNF therapies were used less frequently than they are now, and newer biologic therapies (e.g., ustekinumab and vedolizumab) were not yet available. Additionally, neither study provided a complete picture of the total health system costs of IBD in Canada: The Manitoba Study did not include the costs of ED visits and the Québec study only included the subset of medication costs covered by the provincial government (i.e., medication costs were limited to those for seniors and individuals of lower socioeconomic status). The estimated health system costs in 2018 were likely a substantial underestimate, supported by more recent research on the costs of IBD in Canada (1,33).

The current cost estimates are derived from health administrative data from three Canadian provinces (Alberta, British Columbia, and Manitoba), and then meta-analyzed to provide a pooled per-person cost (7). This cost estimate includes ED data (where available) and medication costs for all individuals, irrespective of their eligibility for provincial pharmacare. Thus, this cost estimate may be more representative of the total IBD costs—though estimates are needed from the remaining seven provinces and all three territories. However, this may still underestimate the current costs of IBD since the proportion of individuals living with IBD on biologic therapy has likely continued to increase since 2017. The growing number of people with IBD, including the increasing number of individuals receiving expensive biologic therapy coupled with low biosimilar uptake, and inflation also contributes to the increased estimate of the total direct healthcare costs in Canada. These increasing costs of IBD are not unique to Canada. Steadily increasing use of biologic therapy has drastically increased the costs of care around the world (4,34,35).

## CONCLUSIONS

Variations and inequities in health services utilization exist across sociodemographic and ethnocultural groups. Since timely access to specialist care is vital for reducing adverse outcomes (e.g., needing to visit the ED), efforts need to focus on ensuring equitable access to high-quality specialist care. Novel models of care for IBD, with the goal of improving outcomes (including reduced reliance on EDs), are described in Mathias et al. (this volume).

Patterns of health services utilization among people living with IBD in Canada are shifting. People with IBD are being admitted to hospitals and undergoing surgery less frequently; this is accompanied by increasing outpatient visits and utilization of biologic therapy. The costs associated with hospitalizations are decreasing (Crohn’s disease) or remaining stable (ulcerative colitis). As the use of expensive biologic therapies continues to increase rapidly, medication costs will eclipse all other health system costs.

The costs of IBD to the healthcare system, people living with IBD, and society are substantial. Over the course of a single year, direct health system and medication costs for IBD are estimated to be $3.33 billion. As the prevalence of IBD continues to rise and as more biologic and targeted therapies at a higher price point than traditional therapies are introduced into the marketplace, the costs of caring for people with IBD will continue to rise (especially through increased costs of prescription medications). Canadian healthcare systems must prepare for the growing number of people with IBD and the costs of caring for these individuals. The costs of therapies need to become more affordable.

## KNOWLEDGE GAPS AND FUTURE RESEARCH DIRECTIONS

1. While we know that there are ethnocultural, sociodemographic and geographic inequities in access to and utilization of the healthcare system by people living with IBD, our knowledge of these inequities concerning tools used to monitor disease proactively (e.g., cross-sectional imaging such as MRI and intestinal ultrasound) is limited. Understanding potential inequities is important for strategies designed to ensure that all individuals living with IBD have timely access to high-quality care that will improve their long-term outcomes.
2. Studies on the health system impacts of biologic therapies have not demonstrated system-level improvements in health services utilization (e.g., hospitalizations and surgeries) and costs of managing IBD. Future studies evaluating the impact of biologic therapy should not only focus on the impact on the healthcare system but on broader society, including the impact of biologic therapies on disability spending, workplace productivity, and other economic benefits resulting from a healthier IBD population.
3. Much of the data describing variation in care for people with IBD comes from regions with relatively high access to specialist care. Data on access to and utilization of the health system among individuals living in rural and remote regions, including the territories, and provinces with limited access to specialist gastroenterology care are sparse and should be a focus of future research.
4. With the emergence of many biosimilars and new competitive advanced therapies (e.g., small molecules, biologics), we should expect that the absolute and relative costs of caring for individuals with IBD will change over time. We need better transparent data to be able to accurately calculate these changing patterns.
5. Our knowledge of the costs of IBD is derived from a subset of provinces. Differences in health system administration are likely a significant source of heterogeneity across provinces. In order to better understand the evolving costs of IBD, national data on the costs of IBD—including the true costs of medications to private and public insurance plans and individuals paying out of pocket for their medications—are needed.

## PATIENT AND CAREGIVER PARTNER PERSPECTIVE

Patient partners underlined that the data presented in this chapter show the distressing picture of the significant direct healthcare costs of IBD (and the inequities). Timely access to outpatient care is essential to prevent negative disease outcomes and healthcare costs. The evidence of IBD healthcare utilization differences depending on where you live, income, or ethnicity is an issue that requires close attention and interventions. For example, rural residents and Firsts Nation individuals with IBD face barriers to accessing primary and specialized care which could result in suboptimal medication management, more emergency department visits, hospitalizations, and surgeries. Improving access to care for individuals with IBD could help reduce healthcare costs in the short and long term. There is an urgency to consider how to support individuals living with IBD to access innovative and holistic healthcare and treatment options. The Canadian healthcare systems need to prepare for the growing number of people with IBD and the costs of caring for them. Since biologic medications account for about 50% of the direct costs of IBD, patient partners also emphasized the need for universal pharmacare in Canada and for developing more research about biosimilars. Upcoming studies and reports could assess the impact of biosimilars on the trends of direct healthcare costs in Canada. The costs of therapies for IBD need to become affordable for individuals, healthcare systems and society overall.

## POLICY IMPLICATIONS AND KEY ADVOCACY OUTCOMES

1. Crohn’s and Colitis Canada should advocate for better regulation of the cost of biologic therapies to ensure the financial viability of providing the right medication to the right person at the right time. Both government and private health insurance plans should provide financial pressure on the pharmaceutical industry to lower medication costs.
2. Despite increasing utilization of cross-sectional imaging that does not expose people living with IBD to ionizing radiation (e.g., MRI, abdominal ultrasound), people living with IBD have limited time access to these imaging modalities. Improving access for people living with IBD to timely imaging for diagnosis and management is important for managing disease before complications arise and will be beneficial for the long-term outcomes of these individuals by reducing costs to individuals, their caregivers, and the healthcare system.
3. Better access to healthcare resources is needed for individuals living in rural and remote communities and in provinces/territories with limited availability of gastroenterologists, including continued use of virtual healthcare beyond the pandemic.
4. Efforts should be made to reduce geographic, ethnocultural, and other sociodemographic inequities in access to and utilization of health services among people living with IBD, particularly high-quality specialist care, which is important for improving the long-term outcomes of IBD.
5. Crohn’s and Colitis Canada should advocate for universal pharmacare that includes timely access to the most effective IBD medications with the goal of improving the short- and long-term well-being of people living with IBD.

## SUPPLEMENT SPONSORSHIP

This article appears as part of the supplement “The Impact of Inflammatory Bowel Disease in Canada in 2023”, sponsored by Crohn’s and Colitis Canada, and supported by Canadian Institutes of Health Research Project Scheme Operating Grant (Reference number PJT-162393).

## Data Availability

No new data were generated or analyzed in support of this review.
